# Application of Polyphenols and Flavonoids in Oncological Therapy

**DOI:** 10.3390/molecules28104080

**Published:** 2023-05-13

**Authors:** Szymon Roszkowski

**Affiliations:** Department of Geriatrics, Collegium Medicum, Nicolaus Copernicus University, Debowa St. 3, 85-626 Bydgoszcz, Poland; szymonr@cm.umk.pl

**Keywords:** natural health products, polyphenols, flavonoids, phytochemicals, cancer, anticancer therapy

## Abstract

The use of naturally derived drugs in anti-cancer therapies has grown exponentially in recent years. Among natural compounds, polyphenols have shown potential therapeutic applications in treatment due to their protective functions in plants, their use as food additives, and their excellent antioxidant properties, resulting in beneficial effects on human health. Building more efficient cancer therapies with fewer side effects on human health can be achieved by combining natural compounds with conventional drugs, which are typically more aggressive than natural chemicals with polyphenols. This article reviews a wide variety of studies where polyphenolic compounds can play a key role as anticancer drugs, alone or in combination with other drugs. Moreover, the future directions of applications of various polyphenols in cancer therapy are shown.

## 1. Introduction

Cancer is a group of diseases that involve the unusual growth of malignant cells with the potential to invade or metastasize to other parts of the body. Lifestyle has a big influence on the causes of cancer and may lead to habits that are fundamental to the development of lifestyle diseases. In addition, pollution, exposure to dangerous chemicals, radiation, stress, smoking, and alcohol consumption can lead to the development of cancer [1]. However, the initiation and development of cancer is not limited to lifestyle causes, but can result from changes in the human genome.

Over the past few years, preclinical and clinical cancer research has identified various collections of developmentally important genes that remain relatively quiescent in normal tissues [2].

Under normal circumstances, the body’s immune system can identify and eliminate cancer cells; however, cancer cells have an “immune escape” mechanism that allows them to evade recognition and attack by the immune system in various ways, allowing them to multiply in the body and prevent elimination [3].

Research to date has made tremendous progress in the prevention, detection, and treatment of cancer, leading to a decline in mortality rates. The use of conventional cancer treatment procedures, such as chemotherapy and radiation therapy, often causes harmful side effects. Therefore, the current goals of cancer research are related to the development of new therapies that are less harmful to the human body. Natural compounds can be very useful in this respect [4,5].

Natural compounds derived from plants or phytochemicals have been used in traditional medicine for centuries. Phytochemicals are chemical compounds that are produced by plants; they are usually involved in the growth of plants or in the process of protecting them against predators or pathogens [6,7]. Currently, the use of phytochemicals, especially polyphenols, as alternative anticancer drugs is a promising alternative to conventional therapies [5,8]. In addition, the human body develops resistance to the conventional drugs that are involved in cancer therapy [9].

Therefore, this article reviews a wide range of polyphenols for their use in different types of cancer therapies.

## 2. Polyphenols

**Figure 1:**
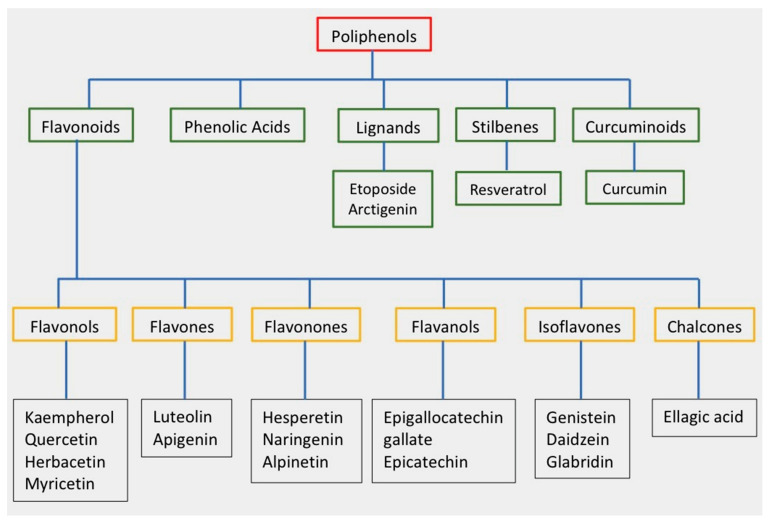
Classification of polyphenols and flavonoids. Examples of each subgroup with antitumor activity are listed.

Polyphenols are secondary metabolites that are produced by plants, and they are characterized by the presence of numerous phenolic rings [10]. The main sources of polyphenols are blueberries, grapes, olive oil, cocoa, nuts, peanuts, and other fruits and vegetables that contain up to 200–300 mg of polyphenols per 100 g fresh weight [11]. Figure 1 shows the classification of the main groups of polyphenols.

Polyphenols can be extracted by simple and ecological techniques, including ultrasonically assisted extraction. T After extraction, polyphenols retain most of their properties. This characteristic facilitates research into the use of these compounds as potential anticancer drugs [12,13].

### 2.1. Flavonoids

The most important group of polyphenols the family. Flavonoids consist of over 6000 molecules that have been identified and isolated. Flavonoids are found in abundance in colorful vegetables and fruits such as blueberries, apples, grapes, oranges, strawberries, plums, and in some common foods and drinks, including dark chocolate, nuts, red wine, tea, soybeans and soybean derivatives, spinach [14].

#### 2.1.1. Flavonols

A rich source of flavonols are many fruits (apples, peaches, oranges, blackberries, and raspberries), vegetables (onions, broccoli, kale, Brussels sprouts, cucumbers, lettuce, tomatoes, potatoes, and spinach), leaves (aloe, rosemary, soybeans, Pinus Sylvestris holly, and endive), seeds (grapes), and grains (several cereals, including quinoa, buckwheat, barley, and oats) [15]. Flavonols are responsible for the color of the flowers of some plants and protect plants against UV and ROS radiation [16].

In addition, flavonols are bioactive polyphenols that are widely used in medicine due to their excellent antioxidant properties as antimicrobial, anti-inflammatory, anti-aging, anticancer, and insecticidal agents. In agriculture, they are commonly used—as pesticides [17].

Kaempferol is a flavonol that is found in plants, plant-based foods, and traditional medicines, including tea, kale, beans, spinach, and broccoli [18]. Several research groups have proven that kaempferol is cytotoxic to breast cancer cells, both in vitro and in vivo [19,20,21,22], inhibiting the growth of neoplastic cells, stopping cell progression and proliferation, and inducing apoptosis of cancer cells.

In separate studies, kaempferol promoted apoptosis of lung cancer cells [23] and exerted an anticancer effect by inhibiting the growth of lung cancer cells and inducing lung cancer cell death [24]. Kaempferol has also been shown to promote the death of cervical cancer cells [25]. Kaempferol also had an obvious regulatory effect on apoptosis of ovarian cancer cells, which indicated that kaempferol may be a promising drug in ovarian cancer [26].

Kaempferol combined with 5-FU exerted a synergistic inhibitory effect on cell viability, enhanced apoptosis, and induced cell-cycle arrest in both chemo-resistant and sensitive colon cancer LS174 cells. Kaempferol also blocked the production of reactive oxygen species (ROS) and modulated the expression of JAK/STAT3, MAPK, PI3K/AKT, and NF-κB signaling in these cells [27].

Quercetin is the most common flavonoid in the human diet [28]. Quercetin is mainly found in red onions, kale, apples, grapes, broccoli, and tea.

Various in vitro and in vivo studies have shown that quercetin is one of the strongest antioxidants of the flavonoid family [29], which makes it an ideal candidate for an anticancer drug. Indeed, quercetin has shown cytotoxicity in various neoplastic cells [30,31].

In breast cancer, the effects of quercetin include modulating the activity of the SOD enzyme, selective inhibition of CYP1B1, CYP2, and CYP3 enzymes, G2/M arrest, and apoptosis [32]. A human breast cancer study showed that quercetin triggers cell death through the mitochondrial and caspase-3 dependent pathways [33,34]. In studies on MCF-7 cells, quercetin not only induced cell cycle arrest, but also induced significant apoptosis [35,36].

Quercetin also induced autophagy and apoptosis in lung cancer cells [37,38] and inhibited lung cancer metastasis [39,40].

Quercetin suppressed genes related to cervical cancer by modulating the epigenetic markers of quercetin [41]. At the same time, quercetin induced apoptosis, leading to the accumulation of ROS [42]. Quercetin inhibited the viability of cervical cancer cells in a dose-dependent manner [43].

In recent studies, quercetin was combined with other drugs to increase the effectiveness of anticancer therapies. Some examples are given below.

In prostate cancer, the combined use of metformin and quercetin exerted significant anti-neoplastic effects via the VEGF/Akt/PI3K pathway [44]. Quercetin directly activated caspase via the mitochondrial pathway, leading to apoptosis in prostate cancer cells [45].

One natural compound that was recently combined with quercetin in cancer therapy research is curcumin. Srivastavaa et al. showed that a mixture of quercetin and curcumin increased the inhibition of cancer cell proliferation by regulating signaling and promoting the death of carcinogenic cells through various pathways [46].

It was also observed that the addition of quercetin to docetaxel in therapy to treat prostate cancer reduced tumor-cell resistance to docetaxel. This increased the effectiveness of neoplastic therapy resulting from the reduction in the proliferation and migration of tumor cells [47].

Quercetin revealed docetaxel resistance-reversing effects in docetaxel-resistant prostate cancer (LNCaP/R, PC-3/R) cells in vitro and in a prostate cancer xenograft model in vivo by reversing the upregulation of P-gp, the development of mesenchymal and stem-like cell phenotypes, and the activation of androgen receptor and PI3K/Akt signaling pathways; moreover, the combinatory treatment of quercetin and docetaxel slowed tumor growth and robustly inhibited proliferation in vivo [48]. Similarly, quercetin enhanced the therapeutic efficiency of paclitaxel in prostate cancer PC-3 cells in vitro through the induction of ER stress and ROS production; this combinatory treatment also exerted beneficial effects in a PC-3 cancer-bearing murine model in vivo [49].

#### 2.1.2. Flavones

Flavones are a class of flavonoids with a chemical structure that is very similar to that of flavonols. They are found mainly in herbs (parsley, thyme, chamomile, mint, and chrysanthemum flowers), and in red or purple plants and vegetables (apple peels, broccoli, cabbage, celery, onion leaves, carrots, and red pepper) [50,51].

Interest in the use of this family of flavonoids in medicine is growing because they exhibit effective antimicrobial, antioxidant, anti-inflammatory, antimutagenic, and anticancer effects [52]. The anticancer properties of apigenin and luteolin have been widely researched.

Luteolin is usually found in the leaves and bark of some plants. The main natural sources of luteolin are celery, thyme, dandelion, clover flower, ragweed pollen, chamomile, and perilla [53].

Due to luteolin’s beneficial effect on the human body (antioxidant and anti-inflammatory properties, supporting carbohydrate metabolism, and modulating the immune system), it is assumed that it may play a role in the treatment of cancer [54,55,56,57].

For example, luteolin decreased the invasive capacity of lung cancer cells [58]. Luteolin showed antitumor activity and reduced cell invasion through Sirt1-mediated apoptosis [59,60].

In two different studies, luteolin reduced breast cancer cell proliferation and induced apoptosis of breast cancer cells [61,62]. The inhibitory effect of luteolin on the invasion of breast cancer cells may be associated with a decrease in VEGF production [63].

In cervical cancer, the expression of some pro-apoptotic genes was increased by luteolin treatment. At the same time, it was also found that the expression of some anti-apoptotic genes was significantly reduced. These results confirmed that luteolin has a strong antiproliferative and pro-apoptotic effect and that this function can probably be achieved by inhibiting the AKT and MAPK pathways [64].

Apigenin is a yellow, crystalline solid. Many fruits and vegetables, such as parsley, celery, celery, carrots, oregano, and chamomile tea, contain apigenin [65,66].

For many centuries, apigenin has been widely used in traditional medicine [67]. The excellent properties of this natural compound prompted research into its use as an anticancer drug [68,69]. Various positive effects of administering apigenin, alone or in combination with other chemotherapeutic agents, have been reported in the literature on various types of cancer treatment [70,71,72]. The following aspects are mentioned: the induction of death of cancer cell lines by triggering both autophagy and apoptosis, the inhibition of migration and the invasion of cancer cells, and the arrest of the cancer-cell cycle.

#### 2.1.3. Flavonones

Flavanones are colorless ketones derived from flavones. Flavanones are found in a wide variety of foods in our daily diet and in herbs [73]. Fruits (especially citrus) that contain flavanones include oranges, lemons, limes, tangelos, and grapefruits. They are also found in strawberries, raspberries, and plums. Vegetables: that contain flavanones include tomatoes and potatoes. Herbs that contain flavanones include rosemary and peppermint.

Flavanones have various functions in plants, including antioxidant functions (pinocembrin) and antimicrobial functions (sakuratenin); they also have taste-modifying properties (eriodictyol, homoeriodictyol, and sterubin).

In recent decades, flavanones have gained significant medical importance due to their antioxidant activity, radical scavenging, cardiovascular effects, anti-inflammatory effects, antiviral effects, and anticancer effects [74]. The most studied as anticancer drugs are hesperetin and naringenin.

Hesperetin and hesperetin 7-O-glycoside (also known as hesperidin) are the main flavonoids found in lemons and sweet oranges [75].

The antitumor properties of hesperetin for specific tumors are well-documented in numerous scientific publications. It improves apoptosis by inducing intracellular ROS formation [76], reduces the activity of NF-κB, which leads to a reduction in tumor progression [77], and inhibits glucose uptake in various tumor cell lines [78].

In a more recent study [79], the addition of hesperetin improved the activity of cisplatin, which is an anticancer drug that is commonly used in the treatment of lung cancer. Hesperetin has been observed to inhibit the multidrug-resistance protein (MDR), which is associated with developed resistance to cisplatin in a large number of patients undergoing anticancer therapy.

In an interesting report, the administration of both naringenin and hesperetin was tested in in vitro and in vivo studies to analyze the antitumor activity in human pancreatic cancer [80]. For the first time, the authors reported that a combination of naringenin and hesperetin could be used as a potential low-toxic anticancer therapy system that inhibits pancreatic cancer development.

In addition to flavonols, the flavanone hesperetin sensitized cisplatin (DDP)-resistant human lung cancer cells (A549/DDP) to cisplatin in vivo and in vitro, mechanistically through the decreased expression of P-gp and the increased intracellular accumulation of the P-gp substrate, rhodamine 123 [81].

Similarly, poncirin, a flavanone glycoside with a bitter taste, enhanced sensitivity to cisplatin by decreasing the expression of MDR-1, MRP1, and BCRP and inhibiting PI3K/Akt signaling in cisplatin-resistant osteosarcoma (OS) cells [82].

Naringenin is the flavanone that is dominant in oranges and grapefruits. It is also found in bergamot, sour oranges, tomatoes, cocoa, water mint, beans, etc. [83,84].

Naringenin has been shown to induce in vitro cytotoxicity in various carcinogenic cells of the breast, stomach, liver, cervix, pancreas, and colon, as well as in leukemia cases [85]. Nevertheless, the poor solubility and instability of naringenin in a physiological environment limits its medical uses. In order to overcome these drawbacks, the synthesis of naringenin derivatives has been proposed. The authors of an earlier study obtained 18 imine derivatives and three alkylated naringenin derivatives that were tested as multidrug resistance (MDR) reversers in cancer cells. While hydrazone and azine derivatives showed an improvement in their MDR-reversal activity against breast-cancer resistance protein, carbohydrazides showed an enhancement of MDR-reversal activity toward a multidrug resistance protein [86]. An alternative recent study showed that naringenin’s efficacy as an anticancer drug in the treatment of breast cancer is due to the activation of the caspase-3 protein and caspase-9 enzymes [87].

Cell-based functional studies showed that naringenin reduces the viability of human cancer cell lines, induces apoptosis, and reduces the cells’ ability to colonize. The results of current in silico and in vitro studies highlighted the importance of naringenin in developing anticancer guidelines for CDK6 inhibitors, with implications for future combinatorial anticancer therapies [88].

#### 2.1.4. Flavanols

Flavanols include catechins and their derivatives. The natural sources of flavanols are mainly the “tea plant” (*Camellia sinensis*) and some cocoa. Therefore, they are often present in the human diet in both beverages (tea) and solid foods (chocolate) [89].

In the research on flavanols in the last few decades, it has been found that these compounds provide resistance to microorganisms, fungi, insects, and herbivores [90,91]. Consequently, the health benefits of flavanols have been extensively researched in humans. Some reports suggest that the consumption of cocoa flavanols may help prevent cardiovascular and metabolic diseases. Indeed, the European Food Safety Authority has approved cocoa products containing 200 mg of flavanols because “they help maintain the flexibility of blood vessels, which contributes to normal blood flow” [92].

Epigallocatechin gallate (3-epigallocatechin gallate or EGCG) is a catechin found mainly in tea. It is one of the polyphenolic compounds that are most commonly found in nature [93]. EGCG has been tested on some cancer-cell lines. In the large intestine HT-29 cell lines, EGCG increased the activity of TfR (transferrin receptor), which is a carrier protein for transferrin, and inhibited the activity of the ferritin-H protein through iron chelating activity in HT-29 colon cancer cells [94].

In another example, the synergistic effect of EGCG and TRAIL (tumor necrosis factor (TNF)-induced apoptosis-inducing ligand), a cell death protein, enhances the activity of both caspase 8 and death receptor 5, resulting in the death of colon-cancer cells SW480 and HCT116 [95].

The correlation between green tea consumption and cancer risk is a well-researched topic [96,97]. For example, a study by Ferrari et al. [98] presented evidence for the modulation of autophagy and antitumor effects induced by EGCG treatment in experimental cancer models. Peer-reviewed papers revealed that EGCG promotes cytotoxic autophagy, often by inactivating the PI3K/Akt/mTOR pathway, inducing apoptosis. It was postulated that the pro-oxidative activity of EGCG is responsible for its antitumor activity. In combination therapy with a chemotherapy drug, EGCG inhibits cell growth and drug-induced survival autophagy. Selected studies rightly claimed that EGCG is a valuable agent in the chemoprevention of cancer.

Despite the fact that EGCG is ubiquitous in nature, this flavanol has some drawbacks that limit its use in cancer therapy (i.e., poor stability, low absorption, and hepatotoxicity) [99]. 

Epicatechin is a flavonoid, large amounts of which are found in cocoa [98]. The use of epicatechin in cancer therapy has recently emerged in an attempt to overcome some of the disadvantages of EGCG [100,101,102].

Pereyra-Vergara et al. investigated the effects and mechanism of action of epicatechin in breast cancer cells [103]. The addition of (−)-epicatechin to carcinogenic cells was shown to cause apoptosis in the two breast cancer cell lines tested (MDA-MB-231 and MCF-7). In addition, the authors showed that (−)-epicatechin increased intracellular ROS production and potentiated the activity of BCL2-related cell death agonist (Bad) and bcl-2-like protein 4 (Bax), proteins that are associated with cell apoptosis.

#### 2.1.5. Isoflavones

Isoflavones are another type of biologically active flavonoid. Isoflavones are found mainly in legume plants (peas, lentils, licorice, beans, and chickpeas), in animal feed (alfalfa, clover, and carob), and as ornamental plants (mimosa and false acacia) [104].

Since isoflavones have estrogenic properties, they are good complementary therapeutic options for treating menopause and its symptoms, such as osteoporosis, anxiety, emotional instability, and headaches. Genistein and daidzein are the most-studied compounds of this subgroup in terms of medical applications.

Genistein is a phytoestrogenic compound produced in soybeans. Genistein has comprehensive biological effects, including anti-diabetic, anti-inflammatory, antioxidant, anti-obesity, and anti-angiogenic effects [105]. The most studied activity is its anticancer activity [106,107].

A number of preclinical and clinical trials of genistein’s antitumor and cytotoxic activity are currently underway in order to develop new therapeutic agents with excellent antitumor potential for the treatment of various types of cancer.

It has been shown that genistein is involved in the regulation of various genes that are associated with the formation of neoplasms through various mechanisms [108].

Moreover, various authors have investigated the effect of genistein in combination with other anticancer drugs [109]. In a recent study, Liu et al. tested mixtures of genistein and cisplatin at various concentrations as a reliable antitumor agent in the treatment of cervical-cancer cells [110]. The authors showed that genistein enhances the antitumor effect of cisplatin and can be used as a chemotherapeutic adjuvant to increase the activity of a chemotherapeutic agent.

#### 2.1.6. Chalcones

Chalcones are a class of polyphenolic compounds that are characterized by the presence of an aromatic ketone and an enone in their central core. Many fruits, such as citrus and apples, and vegetables, such as tomatoes, potatoes, bean sprouts, and some edible plants such as licorice, contain chalcones [111].

The most studied chalcone in the medical field is ellagic acid, which has been studied as a potential anticancer agent [112,113].

Moreover, other classes of flavonoids, such as chalcones, also exhibit potent chemosensitizing capacities in cancer models. The combination of xanthohumol, a prenylated flavonoid from hops, and the chemotherapeutic agent SN38, the active metabolite of irinotecan, in resistant colon cancer SW480 cells decreased cell viability more than SN38 alone was able to do. Therefore, xanthohumol can be potentially utilized as a chemosensitizer of SN38 [114].

Ellagic acid is an antioxidant found in various natural resources, including oak species such as white oak (*Quercus alba*) and red oak (*Quercus robur*) as well as medicinal mushrooms (*Phellinus linteus*). Peaches, pomegranates, grapes, strawberries, raspberries, pecans, walnuts, and raw chestnuts also contain significant amounts of ellagic acid [115].

The antiproliferative and antioxidant properties of ellagic acid have prompted scientists to study the health benefits of this natural compound.

One of the most recent studies evaluating the treatment of breast cancer with ellagic acid was published by Yousuf et al. [116]. In that study, the ability of many phytochemicals other than ellagic acid (capsaicin, tocopherol, limonene, ursolic acid, rosmarinic acid, caffeic acid, and ferulic acid) to inhibit the activity of cyclin-dependent kinase 6 (CDK6), which is associated with cancer progression, was assessed. Of all the natural compounds tested, ellagic acid showed the highest binding affinity for CDK6, reducing tumor proliferation.

In order to improve poor solubility coupled with improved controlled delivery, some research groups have attempted to encapsulate ellagic acid [117,118]. In a recent study, Pirzadeh-Naeeni et al. reported on the nanoencapsulation of ellagic acid in two different biopolymers (schizophilic and chitin), which were then tested on MCF-7 breast-cancer cells [119]. In that case, the controlled release of ellagic acid improved cytotoxicity and reducing tumor cell progression, compared to non-encapsulated ellagic acid.

Other authors [120] investigated the synergistic actions of 17 flavonoids from *Sophora alopecuroides* L. (which is traditionally used as a Chinese herbal medicine) combined with SF against HCC cell lines, together with their primary mechanism. In the experiment, most compounds were found to prominently enhance the inhibitory effects of sorafenib on advanced hepatocellular carcinoma cells more than lone treatments. Among them, three compounds, leachianone A (1), sophoraflavanone G (3), and trifolirhizin (17), exhibited significantly synergistic anticancer activities against MHCC97H cells. Importantly, compounds 3 or 17 combined with sorafenib could synergistically induce MHCC97H cells apoptosis via the endogenously mitochondrial-mediated apoptotic pathway, involving higher Bax/Bcl-2 expressions with the activation of caspase-9 and caspace-3, and arrest the cell cycle in G1 phases. 

Incorporating flavonoids into nanoparticles can confer in situ drug delivery to tumor tissue and manifest enhanced drug accumulation at the tumor site, reducing systemic toxicity, preventing damage to the vascular endothelium, and decreasing the drug administration frequency. Phytosomes and liposomes are the class of nanoplatforms that are currently being extensively used in the market for the delivery of drugs. Phytosomes are the phyto-phospholipid complexes formed by electrostatic interactions between the phospholipid and the phytochemical. They are widely used to specifically deliver herbal bioactives [121].

Several nanoplatforms, such as phytosomes, liposomes, solid lipid nanoparticles, nanocapsules, nanoemulsions, polymeric nanoparticles, lipid-based nanoparticles, and metal-based nanoparticles have been used as delivery vehicles to encapsulate polyphenols for cancer therapy [122].

Nanostructures can be employed to modulate epithelial–mesenchymal transition or to deliver therapeutic molecules against EMT-related pathways.

The application of these functional nanomaterials results in apoptosis induction, the inhibition of cell invasion and migration, increased chemosensitivity, and therapeutic efficacy.

The results of studies to date suggest that nanoparticle-based EMT-inhibition strategies can interfere with various stages of tumor transformation, providing an effective therapeutic approach in the fight against cancer [123]. 

### 2.2. Phenolic Acids

Another subgroup of polyphenols that can be found in several plants, especially in dried fruit, is phenolic acids. These compounds are characterized by the presence of a phenolic ring and have the function of an organic carboxylic acid [124].

Phenolic acid (p-coumaric acid) has shown medicinal properties that make it a likely candidate for the treatment of cancer.

*P-coumaric acid* (or 4-hydroxycinnamic acid) is an organic compound that is derived from cinnamic acid, which can be found in many different edible plants (tomatoes, carrots, garlic, mushrooms, white beans, and other plants). Moreover, p-coumaric acid that is contained in pollen is a component of honey [125].

In the last decade, several studies have been published that confirm the antitumor activity of p-coumaric acid in breast and gastric cancer cells [126,127,128].

### 2.3. Lignans

Lignans are diphenolic compounds found in a wide variety of plants, including broccoli, beans, soybeans, rye, sesame seeds, pumpkin seeds, linseed, and some berries in very small amounts [129]. Lignans are one of the two main groups of phytoestrogens that are known for their own good antioxidant properties [130]. Numerous lignans can be considered possible anticancer drugs. Among them are etoposide, arctigenin, and magnolol, the main lignans that are studied in medicine. In addition, etoposide is a commercial lignan, belonging to the podophyllotoxin subfamily, that is used to treat various types of cancer such as lung cancer and breast cancer [131,132]. However, etoposide chemotherapy has several side effects, including low blood cell counts, vomiting, diarrhea, fever, loss of appetite, and alopecia.

Arctigenin Some plants produce arctigenin, especially in the seeds of the greater burdock (*Arctium lappa*). Current studies have shown that arctigenin inhibits the growth of various cancer cells, including cells in the stomach, lung, liver, and colon, as well as leukocytes [133]. At the same time, the addition of arctigenin enhances the action of caspase-3, a protein that plays a key role in the death of cancer cells. Huang et al. showed that the treatment of OVCAR3 and SKOV3 ovarian cancers with arctygenin causes apoptosis of neoplastic cells in vitro [134].

Lee et al. investigated the effect of arctigenin (ATG) on doxorubicin-induced cell death (DOX) using human breast cancer cells MDA-MB-231. The results showed that DOX-induced cell death was enhanced by concurrent treatment with ATG/DOX in a concentration-dependent manner and that this was associated with increased DOX uptake and the suppression of multidrug-resistance-associated protein 1 (MRP1) gene expression in MDA-MB cells-231 [135].

### 2.4. Resveratrol

Resveratrol is a natural polyphenol from the stilbene family. Resveratrol is produced by several plants (grapes, almonds, beans, blueberries, raspberries, mulberries, peanuts, etc.) in response to infections and injuries, or as a defense against various attacks by pathogens such as fungi and bacteria [136]. In addition, red wine contains significant amounts of resveratrol.

In 1997, Jang et al. were the first investigators to report the inhibition of skin cancer development in mice with resveratrol [137]. Since then, many studies have suggested that resveratrol is able to prevent or delay the onset of cancer [138,139,140].

In fact, studies have shown that resveratrol is active in vitro against many human cancers, including cancers of the breast, skin, ovary, stomach, prostate, colon, liver, pancreas, and cervix, as well as thyroid cancer cells, lymphoid carcinoma cells, and myeloid carcinoma cells [141].

Resveratrol has been shown to be beneficial at various stages of neoplastic disease (tumor initiation, promotion, and progression). For example, resveratrol protects DNA against reactive oxygen species (ROS) and traps the hydroxyls, superoxides, and free radicals produced in cells (events that are usually associated with tumor initiation) [142].

In addition, human clinical trials with resveratrol have been conducted with satisfactory results [143,144,145].

### 2.5. Curcuminoids

Curcuminoids are natural polyphenols that contain two phenolic units linked by a linear diarylheptanoid. Among them, curcumin is one of the best known and researched structures, with high potential as a drug. Nevertheless, the poor solubility of curcumin in water of acidic and physiological pH requires a variety of alternatives to avoid losing the efficacy of curcumin as a drug [146].

Curcumin has been used in anticancer therapies for various types of cancer: lung, cervical, prostate, breast, bone, and liver [147]. Nevertheless, the administration of free curcumin has some drawbacks, including poor water solubility, instability under water conditions, and low bioavailability [148].

Many different clinical trials have been conducted on the use of curcumin as an anticancer drug. Recently, various research groups have reported that the combination of curcumin with gemcitabine-based chemotherapy is safe and that its use is possible in pancreatic cancer patients [149,150,151].

Overall, gemcitabine adjuvant therapy with curcumin phytosome complex is not only safe, but it also effectively translates into a good first-line response rate for advanced pancreatic cancer.

Table 1 shows the most important studies on the molecular mechanisms of anticancer activity of the main polyphenols and flavonoids.

## 3. Clinical Potential of Flavonoids

Flavonoids have the potential to improve treatment outcomes for several types of cancer. However, we still need to develop our understanding of the molecular mechanisms by which flavonoids act as anticancer drugs. Preclinical studies have identified several flaws in flavonoids that hinder their anticancer properties when administered systemically. 

Although it has been preliminarily confirmed in vivo and in vitro that the combination of flavonoids and chemotherapeutic drugs plays a role through targeted autophagy, clinical trial data are currently insufficient.

Increasing our understanding of nanotechnology-based drug delivery has shown that it can improve outcomes for several types of cancer. Flavonoid-filled nanoparticles may be more beneficial than native flavonoids, acting as new therapeutic agents in the treatment of cancer. Thus, the use of functional nanomaterials results in the induction of apoptosis, the inhibition of cell invasion and migration, and the increase in chemosensitivity and therapeutic effectiveness.

Despite these benefits, it is important to remember that, in some cases, nanomaterials can overstimulate healthy tissues, leading to dangerous effects, including inflammation and fibrosis. However, the available evidence is based on preclinical studies conducted in small animal models, which do not answer the broader questions of their translation to clinics for direct benefit to humans. The use of nanotechnology to deliver flavonoids faithfully reflects the ability to transfer them from preclinical settings to clinical trials.

## 4. Conclusions

Overall, the unique comprehensive biological effects of polyphenols, including anti-inflammatory, antioxidant, and anti-angiogenic effects, make them strong candidates for a variety of cancer treatments. In fact, the antitumor activity of several polyphenolic compounds has been studied, mainly in tumor cells in vitro and in preclinical animal models.

Nevertheless, there is very little clinical data covering the most common polyphenols, such as resveratrol, curcumin, and quercetin, for use as anticancer drugs.

Research on cancer therapies, especially the use of flavonoids, has led to the development of natural drugs that are less aggressive than conventional cancer drugs. In fact, various research studies have shown that polyphenols can be used as adjuvants to chemotherapy.

However, the process of discovering the interactions of polyphenols with cancer and their mechanisms of action requires further research in order for these natural compounds to improve actual anticancer strategies.

## Figures and Tables

**Table 1:** Mechanisms of anticancer activity of selected polyphenols and flavonoids.

Flavonoids	Cancer Model	Mechanisms	Ref
Apigenin	Hepatocellular carcinoma doxorubicin-resistant cell BEL-7402/ADM Nude mice	Sensitizes drug-resistant cells to doxorubic through suppressing miR-520b/ATG7 axis.	[152]
	Breast cancer T47D, MDA-MB-231	Induction of protective autophagy and apoptosis.	[153]
	Colorectal cancer HCT116	Autophagy inhibitor significantly enhanced the apoptosis.	[154]
	Hepatocellular carcinoma Hepg2	Increases levels of Caspase-3, PARP cleavage, and Bax/Bcl-2 ratios.	[155]
	Non-small cell lung cancer EGFR-TKIs-resistant NCI-H1975 (Apigenin + Gefitinib)	Inhibits the AMPK pathway and autophagy flux, leading to enhanced apoptotic cell death. Inhibits multiple oncogenic drivers such as c-Myc, HIF-1α, and EGFR, and reduces Gluts and MCT1 protein expression. Downregulates Cyclin D1, CDK4, E-cadherin, MMP2, and MMP9, and induces G0/G1 cell cycle arrest and cell metastasis.	[156]
	Colorectal cancer cisplatin-resistant cell HT-29	Induces autophagic cell death and inhibits the growth of cells by targeting the m-TOR/PI3K/AKT signaling pathway. Autophagy inhibits the occurrence of MDR.	[157]
	Breast cancer (MDA-MB-468), prostate cancer (PC3),	The investigated compounds cause intracellular copper mobilization and ROS production, resulting in cancer cell death.	[158]
Baicalein	Prostate cancer PC-3, DU145 Breast cancer MDA-MB-231	Activation of AMPK and ULK1 and downregulation of mRNA level of mTOR/Raptor induces autophagic cell death. Upregulates the expression of Beclin1, Atg5, Atg7, ULK1, and LC3B-II. Induction of autophagic cell death.	[159]
	Breast cancer MCF-7, MDA-MB-231	Induces apoptosis and autophagy by inhibiting the PI3K/AKT pathway.	[160]
	Non-small cell lung cancer A549, H1299	Induces the loss of mitochondrial membrane potential and the release of cyto-c and apoptosis inducing factor into the cytoplasm. Induces autophagy and activates autophagy flux.	[161]
	Human glioblastoma U87 and U251 cell lines	Maturation of microtubule-associated protein 1A/1B-LC3B indicated the activation of autophagy potentially through the PI3K/Akt/mTOR pathway, and inhibition of autophagy by 3-methyladenine decreased the apoptotic cell ratio.	[162]
Quercetin	Glioblastoma multiforme T98G (quercetin + temozolomide) Anaplastic astrocytoma MOGGCCM (quercetin + temozolomide)	Activates ER stress, increases the level of caspase 12 expression, and changes the shape of nuclei. Inhibition of HSP expression results in severe apoptosis and no obvious signs of autophagy, which decreases mitochondrial membrane potential, and increases level of cyto-c in the cytoplasm and the activation of caspase 3 and caspase 9.	[163]
	Glioblastoma U251, U87	T-AUCB induces overexpression of Atg7 and regulates autophagy-related gene expression.	[164]
	Glioblastoma multiforme T98G (quercetin + sorafenib)	In T98G cells, sorafenib mainly initiated autophagy, resulting in an increased number of autophagic cells with quercetin.	[165]
	Glioblastoma U373MG	Activates JNK signal, increases the expression and translocation of p53 to the mitochondria, and causes the release of cyto-c into the cytoplasm.	[166]
	Melanoma (B16-F10)	Inhibits Akt/PI3 K and MEK-ERK signaling while augmenting UVB-induced nuclear translocation of NF-*κ*b.	[167]
Galangin	Laryngeal carcinoma TU212, HEP-2	Modulates apoptosis through caspase-3, caspase-9, and PARP cleavage activation and bcl-2 downregulation. Regulates apoptosis and autophagy by p38 and AKT/NF-κB/mTOR pathways.	[168]
*Epigallocatechin* *gallate*	Non-small cell lung cancer A549 (gefitinib-resistant cell)/	Inhibits autophagy induced by gefitinib and promotes cell death.	[169]
	Colorectal cancer HCT-116	The combined effect of epigallocatechin Gallate and quercetin caused cell cycle arrest at the G1 phase.	[170]
Chalcone	Breast cancer Epirubicin-resistant cell MCF-7/ADR	Induction of autophagy and G2/M checkpoint block and downregulation of ABCG2 expression, but no induction of apoptosis. Induces autophagic cell death through inhibition of miR-25 and upregulation of ULK1 expression.	[171]
	Breast cancer MCF-7 cells	Licochalcone A inhibits PI3K/Akt/mTOR activation and promotes autophagy and apoptosis in MCF-7 cells	[172]
	Malignant melanoma	Cell-cycle arrest at the G2/M phase was associated with modulation of expression or phosphorylation of specific cell cycle-associated proteins (cyclin B1, p21, and ChK1) and tubulins.	[173]
	*Human uterine sarcoma*	Induces A375 cells to differentiate and lose their pluripotency by inhibiting the expression of Notch1, β-catenin, and Oct-3/4 and targeting members of the key signals PI3K/Akt and MEK-ERK pathways.	[174]
	Ovarian cancer OVCAR5 and ES-2	Isoliquiritigenin induced G2/M phase arrest. Furthermore, the expression of cleaved PARP, cleaved caspase-3, Bax/Bcl-2 ratio, LC3B-II, and Beclin-1 levels were increased in Western blot analysis.	[175]
	Human breast cancer	Cell-cycle arrest at G2/M phase and induced apoptosis and autophagy in human breast cancer cells. Interruption of the PI3K/AKT/mTOR/p70S6K/ULK signaling pathway.	[176]

## Data Availability

Publicly available datasets were analyzed in this study.
